# Dissecting the Molecular Mechanism of the Subcellular Localization and Cell-to-cell Movement of the *Sugarcane mosaic virus* P3N-PIPO

**DOI:** 10.1038/s41598-017-10497-6

**Published:** 2017-08-29

**Authors:** Guangyuan Cheng, Meng Dong, Qian Xu, Lei Peng, Zongtao Yang, Taiyun Wei, Jingsheng Xu

**Affiliations:** 1 Key Laboratory of Sugarcane Biology and Genetic Breeding, Ministry of Agriculture, Fujian Agriculture and Forestry University, Fuzhou, 350002 Fujian, China; 2 State Key Laboratory of Ecological Pest Control for Fujian and Taiwan Crops, Institute of Plant Virology, Fujian Agriculture and Forestry University, Fuzhou, 350002 Fujian, China

## Abstract

The coding sequence of P3N-PIPO was cloned by fusion PCR from *Sugarcane mosaic virus* (SCMV), a main causal agent of sugarcane (*Saccharum* spp. hybrid) mosaic disease. SCMV P3N-PIPO preferentially localized to the plasma membrane (PM) compared with the plasmodesmata (PD), as demonstrated by transient expression and plasmolysis assays in the leaf epidermal cells of *Nicotiana benthamiana*. The subcellular localization of the P3N-PIPO mutants P3N-PIPOT1 and P3N-PIPOT2 with 29 and 63 amino acids deleted from the C-terminus of PIPO, respectively, revealed that the 19 amino acids at the N-terminus of PIPO contributed to the PD localization. Interaction assays showed that the 63 amino acids at the C-terminus of PIPO determined the P3N-PIPO interaction with PM-associated Ca^2+^-binding protein 1, ScPCaP1, which was isolated from the SCMV-susceptible sugarcane cultivar *Badila*. Like wild-type P3N-PIPO, P3N-PIPOT1 and P3N-PIPOT2 could translocate to neighbouring cells and recruit the SCMV cylindrical inclusion protein to the PM. Thus, interactions with ScPCaP1 may contribute to, but not determine, SCMV Pm3N-PIPO’s localization to the PM or PD. These results also imply the existence of truncated P3N-PIPO in nature.

## Introduction

The movement of a virus from an infected cell to an adjacent cell through the plasmodesmata (PD) is an important step in establishing a systemic infection in a host^1, 2^. PD are plasma membrane (PM)-lined channels that traverse the cell walls between adjacent cells, gating the symplastic movement of transcription factors, mRNAs and siRNAs, as well as pathogenic intruders^3^. Small molecules diffuse through PD, while larger objects, such as plant viruses, have to increase the size exclusion limit (SEL) of PD to traffic between cells^3–5^. Plant viruses have evolved different types of movement proteins (MPs) to modify PD gating properties to allow virus or viral nucleic acid movement^1, 2, 6^. For example, members of the genus *Tobamovirus* encode a single 30-kD dedicated MP to transport viral genomes through the modified PD^7–9^. Members of the genus *Nepovirus* and the genus *Comovirus* encode MPs to form tubular structures that mediate virion cell-to-cell movement^10, 11^. Members of genus *Potexvirus*, genus *Hordeivirus* and genus *Pomovirus* possess a triple gene block encoding three MPs that act cooperatively to promote movement of the viral complex^2, 12–14^. The MP remains unknown for the genus *Potyvirus* in the family *Potyviridae*, which consists of approximately 30% of known plant viruses.

The genomes of *Potyvirus* members consist of single-stranded, positive-sense RNAs of approximately 10,000 nucleotides that encode single large polyproteins which self-cleave to yield 10 mature proteins, which are: P1, helper component proteinase (HC-Pro), P3, 6K1, cylindrical inclusion (CI), 6K2, viral genome-linked protein (VPg), proteinase domain of NIa (NIa-Pro), nuclear inclusion protein b, and coat protein (CP)^15, 16^. Another protein, named P3N-PIPO, is a recently identified fusion protein containing the N-terminal regions of P3 and PIPO, embedded within the P3 cistron, but translated from an RNA template generated by transcriptional slippage with a +1 A insertion in the motif GAAAAAA^17–19^. Among these 11 proteins, CP, CI, HC-Pro, VPg, 6K2 and P3N-PIPO are involved in viral cell-to-cell movement^20–25^. Accumulating evidence suggests that P3N-PIPO functions as an MP in *Potyviridae* ^17, 21, 22, 26–28^. For example, synonymous mutations in the P3 cistron that altered the *pipo* open reading frame (ORF) of *Wheat streak mosaic virus* disrupted the virus movement in plants^28^. Accordingly, introducing premature stop codons within *pipo* or mutations in the conserved G_1–2_A_6–7_ motif without altering the P3 amino acid sequence in the *Soybean mosaic virus* (SMV) genome restricted viral cell-to-cell movement^27^.

*Sugarcane mosaic virus* (SCMV; *Potyvirus* genus in the *Potyviridae* family) is a main pathogen causing severe mosaic disease, which results in yield loss, in sugarcane (*Saccharum* spp. hybrid), the most important sugar and energy crop worldwide^29, 30^. However, there is limited information on the mechanism of SCMV infection in sugarcane^31^. Here, we present evidence that SCMV P3N-PIPO localizes preferentially to the PM compared with the PD. A series of truncated P3N-PIPO mutants were generated to map the regions responsible for subcellular localization and the regions responsible for interaction with the sugarcane PM-associated Ca^2+^-binding protein 1, ScPCaP1, isolated from sugarcane. The truncated P3N-PIPO with 63 amino acids deleted from the C-terminus of PIPO localized to PD, with some aggregates in the cytoplasm, independent of interactions with ScPCaP1. This truncated P3N-PIPO and the one with 29 amino acids deleted from the C-terminus of PIPO could move to neighbouring cells and recruited CIs to PM, like the wild-type SCMV P3N-PIPO.

## Results

### SCMV P3N-PIPO was preferentially localized to the PM compared with the PD

The P3 cistron was cloned from SCMV by RT-PCR, and its sequence was deposited into GenBank (accession number KY379821). The motif GA_6_ was found in the P3 cistron. Fusion PCR was applied to obtain the *P3N-PIPO* coding sequence. *P3N-PIPO* is 708 nt in length, encoding a 235-amino acid peptide, which contains 153-amino acid P3N and 82-amino acid PIPO.

**Figure 1:**
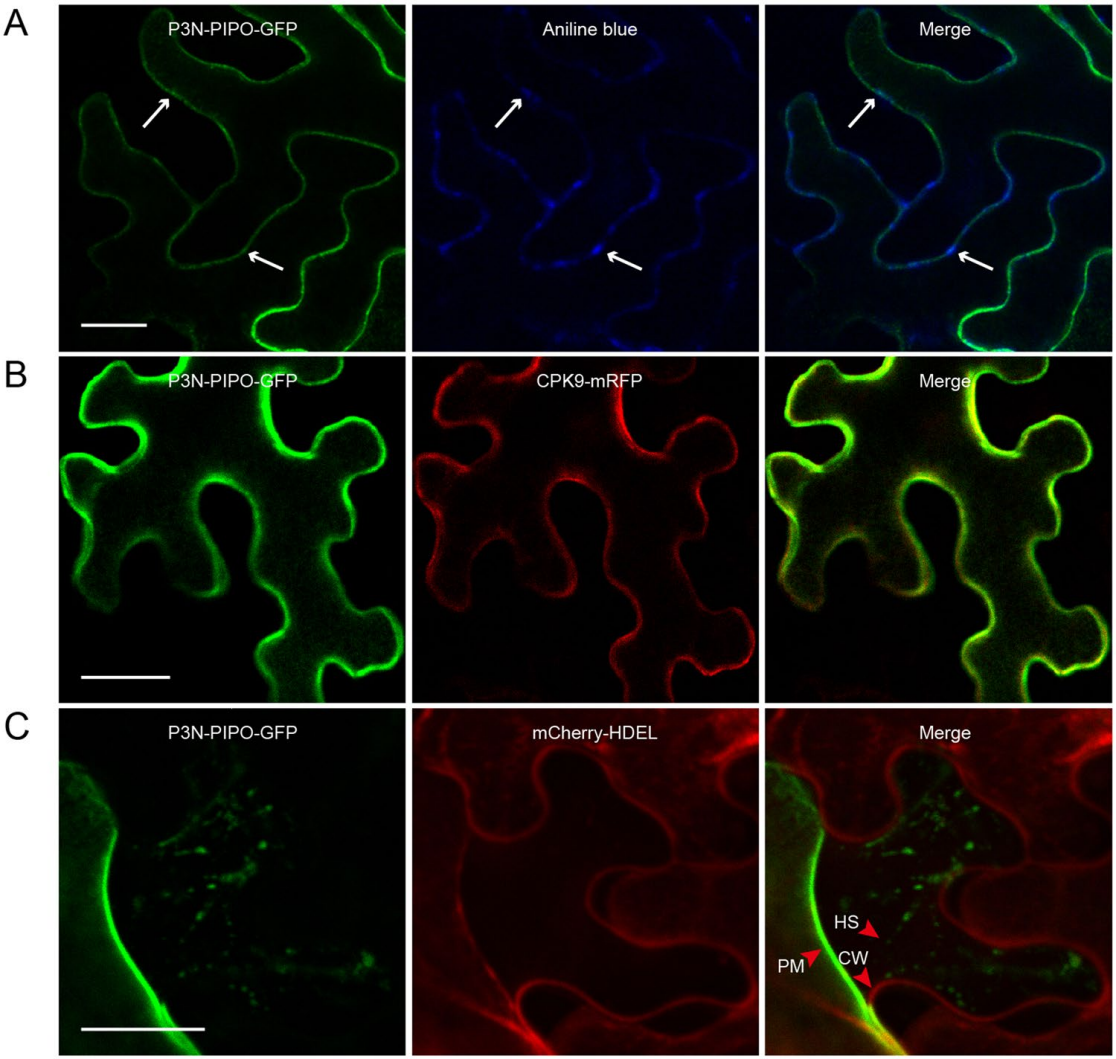
Subcellular localization of SCMV P3N-PIPO in *N*. *benthamiana* leaf epidermal cells. (**A**) Fluorescence of P3N-PIPO-GFP detected on PM by 48 h post agroinfiltration (hpa). Callose staining with aniline blue showing PD sites. Merged image showing that P3N-PIPO localizes to the PM and PD. White arrows point to PD. (**B**) Localization of P3N-PIPO-GFP in leaf cells expressing the PM marker CPK9-mRFP by 48 hpa. (**C**) Localization of P3N-PIPO-GFP in leaf cells expressing endoplasmic reticulum marker mCherry-HDEL under plasmolysis treatment. CW, cell wall. HS, Hechtian strands. PM, plasma membrane. Scale bars, 25 μm.

To determine the subcellular localization of SCMV P3N-PIPO, P3N-PIPO-green fluorescent protein (GFP) was expressed in *Nicotiana benthamiana* leaves by agroinfiltration. Leaves were infiltrated with aniline blue and imaged by confocal laser scanning microscopy^32^. Fluorescence from P3N-PIPO-GFP overlaid fluorescence due to aniline blue, forming several punctate structures at the PD with abundant bright green fluorescence along the PM (Fig. 1A). The SCMV P3N-PIPO appears to preferentially localize to the PM. When SCMV P3N-PIPO was co-expressed in *N*. *benthamiana* leaf cells with the PM localization protein CPK9-mRFP^33^, the green fluorescence of P3N-PIPO-GFP overlapped with the red fluorescence of CPK9-mRFP, indicating that SCMV P3N-PIPO localized to the PM (Fig. 1B). To further confirm the SCMV P3N-PIPO PM localization, P3N-PIPO-GFP was co-expressed with mCherry-HDEL, which is an endoplasmic reticulum marker protein, and was then subjected to plasmolysis treatment. The green fluorescence of P3N-PIPO-GFP remained on the PM and overlapped with the red fluorescence of mCherry-HDEL when the cytoplasm detached from the cell wall (Fig. 1C). The green fluorescence of P3N-PIPO-GFP was observed on Hechtian strands between the PM and cell wall (Fig. 1C). Thus, we concluded that SCMV P3N-PIPO was predominantly localized to the PM *in N*. *benthamiana*.

### The 19-amino acid domain at the N-terminus of PIPO conditions SCMV P3N-PIPO localization to the PD

**Figure 2:**
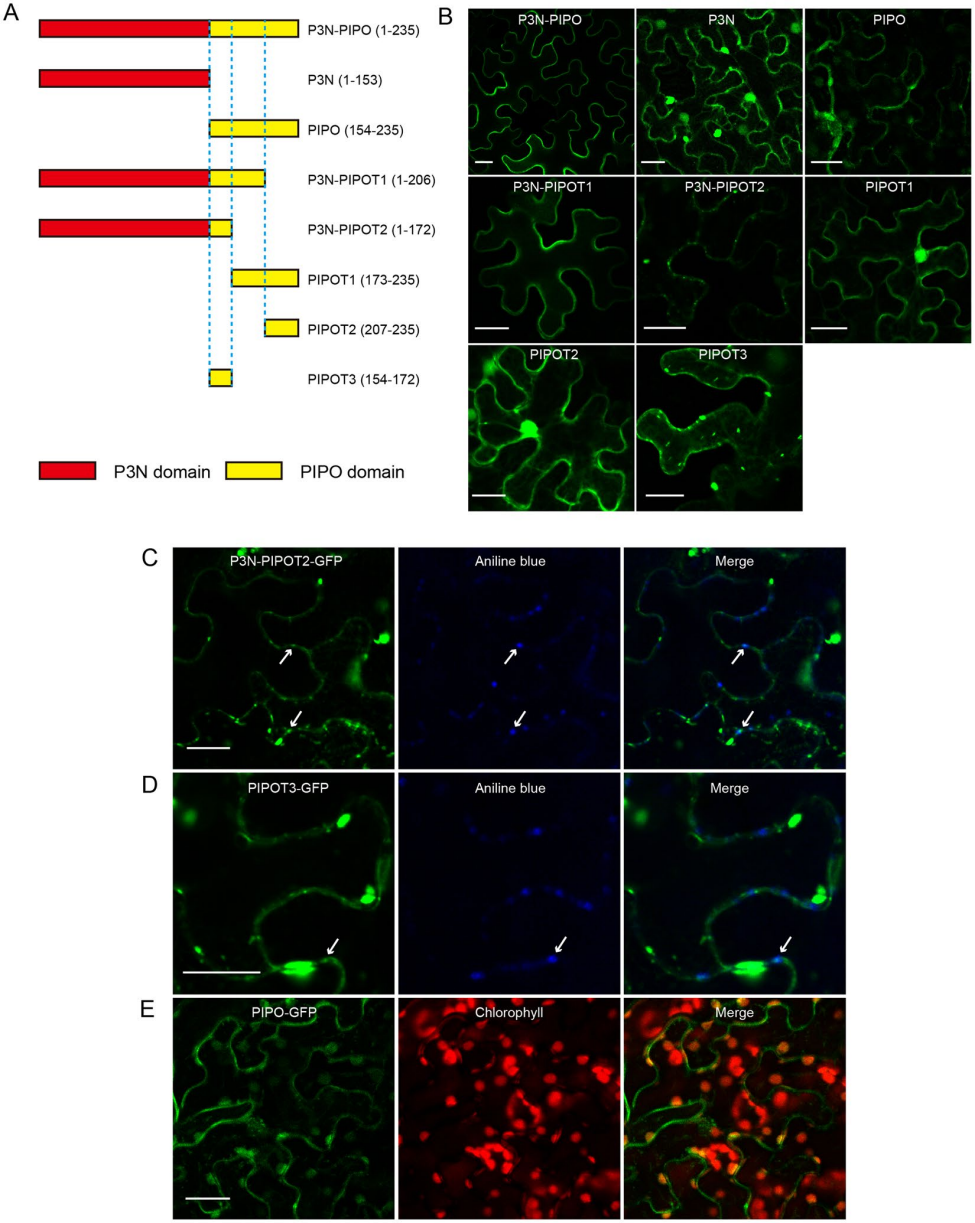
Subcellular localization of truncated variants of P3N-PIPO in *N*. *benthamiana* leaf epidermal cells. (**A**) Diagram representation of wild-type and truncated P3N-PIPO fragments. Red boxes represent P3N domains, yellow boxes represent the full-length and truncated PIPO domains of different lengths, and imaginary lines indicate the boundaries of different sections. (**B**) Transient expression of truncated P3N-PIPO fragments fused with GFP in *N*. *benthamiana* leaves. Image shows that P3N-PIPOT2-GFP formed punctate structures on the PM. (**C**) Localization of P3N-PIPOT2-GFP with PD marker aniline blue by 48 hpa. Arrows point to PD. (**D**) Localization of P3N-PIPOT3-GFP with PD marker aniline blue by 48 hpa. Arrows point to PD. (**E**) Co-localization of PIPO-GFP with chloroplasts by 48 hpa. Scale bars, 25 μm.

The ambiguous subcellular localization of SCMV P3N-PIPO led us to identify the domain responsible for the PM or PD association. A series of truncated P3N-PIPO mutations were produced. The truncated P3N-PIPO fragments (P3N-PIPOT1, P3N-PIPOT2, P3N, PIPO, PIPOT1, PIPOT2 and PIPOT3), as illustrated in Fig. 2A, were fused with GFP. These constructs were transiently expressed in *N*. *benthamiana* leaves independently through agroinfiltration. The green fluorescence of GFP was observed by 48 h post agroinfiltration (hpa). Compared with the wild-type SCMV P3N-PIPO, the truncated P3N-PIPOT1 with 29 amino acids deleted at the C-terminus of P3N-PIPO, showed PM localization (Fig. 2B). However, the truncated P3N-PIPOT2 with 63 amino acids deleted at the C-terminus of P3N-PIPO showed punctate structures on the PM indicating its PD localization (Fig. 2B). To further confirm the PD localization of P3N-PIPOT2, we used aniline blue to stain the callose in the *N*. *benthamiana* epidermal cells^32^. The green fluorescence of P3N-PIPOT2-GFP merged with the blue fluorescence of aniline (Fig. 2C). The PIPO’s C-terminal 63 amino acid domains in P3N-PIPO appeared to contribute to PM localization because P3N alone showed no specific localization (Fig. 2B). However, PIPOT1 (the C-terminal 29 amino acid domain of PIPO) and PIPOT2 (the C-terminal 63 amino acid domain of PIPO) showed no specific subcellular localizations in the *N*. *benthamiana* epidermal cells (Fig. 2B), whereas PIPOT3 (the N-terminal 19 amino acid domain of PIPO) could localize to PD and aggregate in the cytoplasm (Fig. 2B,D). Interestingly, the full-length PIPO was localized not only to the PD but also to chloroplasts (Fig. 2B,E). Thus, we conclude that the 19-amino acid domain of the N-terminus of PIPO contributes to the localization of SCMV P3N-PIPO to the PD.

### PM or PD targeting of SCMV P3N-PIPO requires the early secretory pathway and is independent of the actin cytoskeleton

Latrunculin B (Lat B) and Brefeldin A (BFA) interfere with cellular processes and have been used to investigate viral intracellular trafficking^21, 26^. Lat B is a chemical inhibitor of actin polymerization^34^. BFA impairs the vesicular transport between the endoplasmic reticulum and Golgi^35^. In this study, leaves of *N*. *benthamiana* were treated with Lat B and BFA independently and were observed by confocal microscopy. The localization of SCMV P3N-PIPO to the PM or the PD was independent of the actin cytoskeleton, while intracellular trafficking required the early secretory pathway (Supplementary Fig. S1). These findings are consistent with previous reports on the P3N-PIPO of *Turnip mosaic virus* (TuMV), *Tobacco etch virus* (TEV) and *Tobacco vein banding mosaic virus* (TVBMV)^21, 26^.

### ScPCaP1 interacts with SCMV P3N-PIPO

PCaP1 was identified as a host factor playing key roles in TuMV and TVBMV infections in hosts through interactions with P3N-PIPO^22, 26^. The interactions between TuMV P3N-PIPO and *Arabidopsis thaliana* PCaP1 are determined by the PIPO domain, but not by the P3N domain^22^. The interactions between TVBMV P3N-PIPO and NbDREPP, a homologue of AtPCaP1, are determined by a 17-amino acid domain of the C-terminus of PIPO^26^. In this study, sugarcane *PCaP1* was cloned from the sugarcane cultivar *Badila* and designated *ScPCaP1* (GenBank accession number KY379820). The *ScPCaP1* ORF is 702 bp, encoding a 233-amino acid protein with a conserved N-terminus and an intrinsically disordered C-terminus.

**Figure 3:**
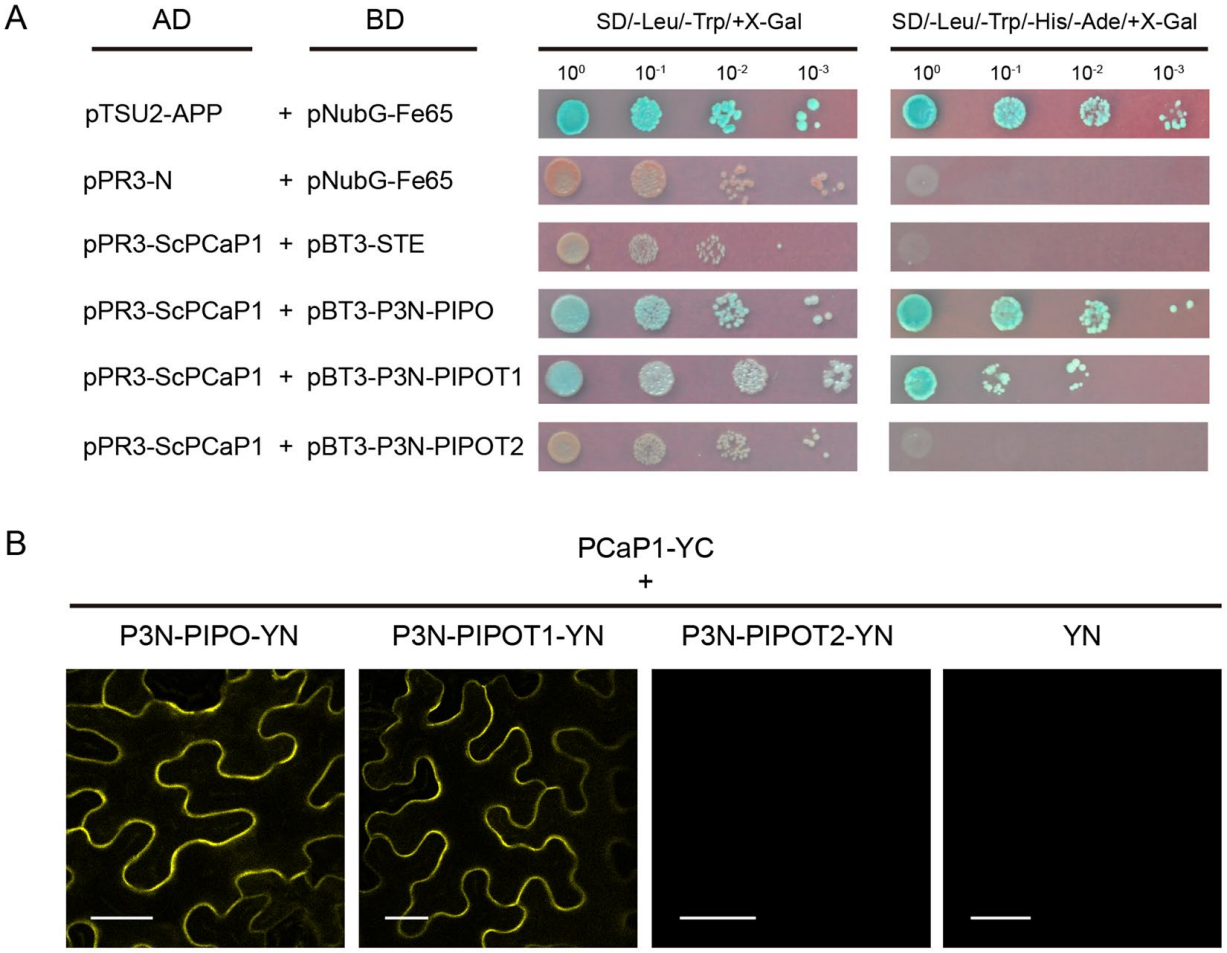
Analysis of the P3N-PIPO domain for binding to ScPCaP1. (**A**) Interactions between ScPCaP1 and wild-type or truncated P3N-PIPOs in yeast cells. Yeast strain *NMY51* co-transformed with the pPR3-ScPCaP1 prey vector in combination with the bait vector pBT3-P3N-PIPO, pBT3-P3N-PIPOT1, pBT3-P3N-PIPOT2, or an empty bait vector and were plated on non-selective medium (SD/−Leu/−Trp/+X-Gal) or a high-stringency selective medium (SD/−Leu/−Trp/−His/−Ade/+X-Gal). pTSU2-APP and pNubG-Fe65 were used as positive controls, while pPR3-N and pNubG-Fe65, and pPR3-ScPCaP1 and pBT3-STE were used as negative controls. (**B**) Bimolecular fluorescence complementation assay of P3N-PIPO, P3N-PIPOT1 and P3N-PIPOT2 with ScPCaP1 in *N*. *benthamiana* leaf cells. Full-length and truncated P3N-PIPO fragments were fused with YN, and ScPCaP1 was fused with YC. YN (empty vector) and ScPCaP1-YC were used as blank controls. The images were taken at 40 hpa. YN, N terminus of YFP; YC, C terminus of YFP. Scale bars, 25 μm.

To determine if ScPCaP1 interacts with SCMV P3N-PIPO, a split ubiquitin-based membrane yeast two-hybrid (Y2H) system (Clontech, Mountain View, CA, USA) was used according to the manufacturer’s protocols. The prey vector pPR3-ScPCaP1 and the bait vectors pBT3-P3N-PIPO, pBT3-P3N-PIPOT1 and pBT3-P3N-PIPOT2 were pairwise co-transformed into the *Saccharomyces cerevisiae* strain *NMY51*. Synthetic dextrose (SD) double dropout (DDO), SD/−Trp/−Leu, agar plates and SD quadruple dropout (QDO), SD/−Trp/−Leu/−His/−Ade, agar plates were used for the yeast cultures. Like the yeast cells co-transformed with the positive control plasmids, the yeast cells co-transformed with pPR3-ScPCaP1 and pBT3-P3N-PIPO, pBT3-P3N-PIPOT1 or pBT3-P3N-PIPOT2 produced blue colonies on the DDO and QDO culture media supplemented with 5-bromo-4-chloro-3-indolyl β-D-galactoside (X-Gal) (Fig. 3A). The plasmid pair pPR3-ScPCaP1 and pBT3-P3N-PIPOT2 and the negative control plasmids showed no interactions (Fig. 3A). The results demonstrated that ScPCaP1 interacts with P3N-PIPO and P3N-PIPOT1, but not with P3N-PIPOT2.

To further confirm the interactions between ScPCaP1 and SCMV P3N-PIPO, and between ScPCaP1 and the truncated P3N-PIPOs, bimolecular fluorescence complementation (BiFC) assays were conducted in *N*. *benthamiana* leaves. SCMV P3N-PIPO, P3N-PIPOT1 and P3N-PIPOT2 were individually fused in frame to the N-terminal half of the yellow fluorescent protein (YFP) (YN) to generate P3N-PIPO-YN, P3N-PIPOT1-YN and P3N-PIPOT2-YN, respectively. ScPCaP1 was fused in frame to the C-terminal half of YFP (YC) to generate ScPCaP1-YC. The YFP-fusion constructs were co-transformed pairwise into *Agrobacterium tumefaciens* strain *EHA105*, then agroinfiltrated into *N*. *benthamiana* leaves. By 48 hpa, the yellow fluorescence of YFP was observed by confocal microscopy in leaf cells co-expressing ScPCaP1-YC and P3N-PIPO-YN or P3N-PIPOT1-YN (Fig. 3B). As expected, no yellow fluorescence was observed in the leaf cells co-expressing ScPCaP1-YC and P3N-PIPOT2-YN. ScPCaP1-YC and YN served independently as negative controls (Fig. 3B). Thus, the BiFC results confirmed that ScPCaP1 could interact with SCMV P3N-PIPO and P3N-PIPOT1, but not with P3N-PIPOT2, in *N*. *benthamiana* leaves. Thus, we concluded that ScPCaP1 interactions with SCMV P3N-PIPO are determined by the C-terminal 63-amino acid domain of PIPO.

### SCMV P3N-PIPO recruits CI to PM

**Figure 4:**
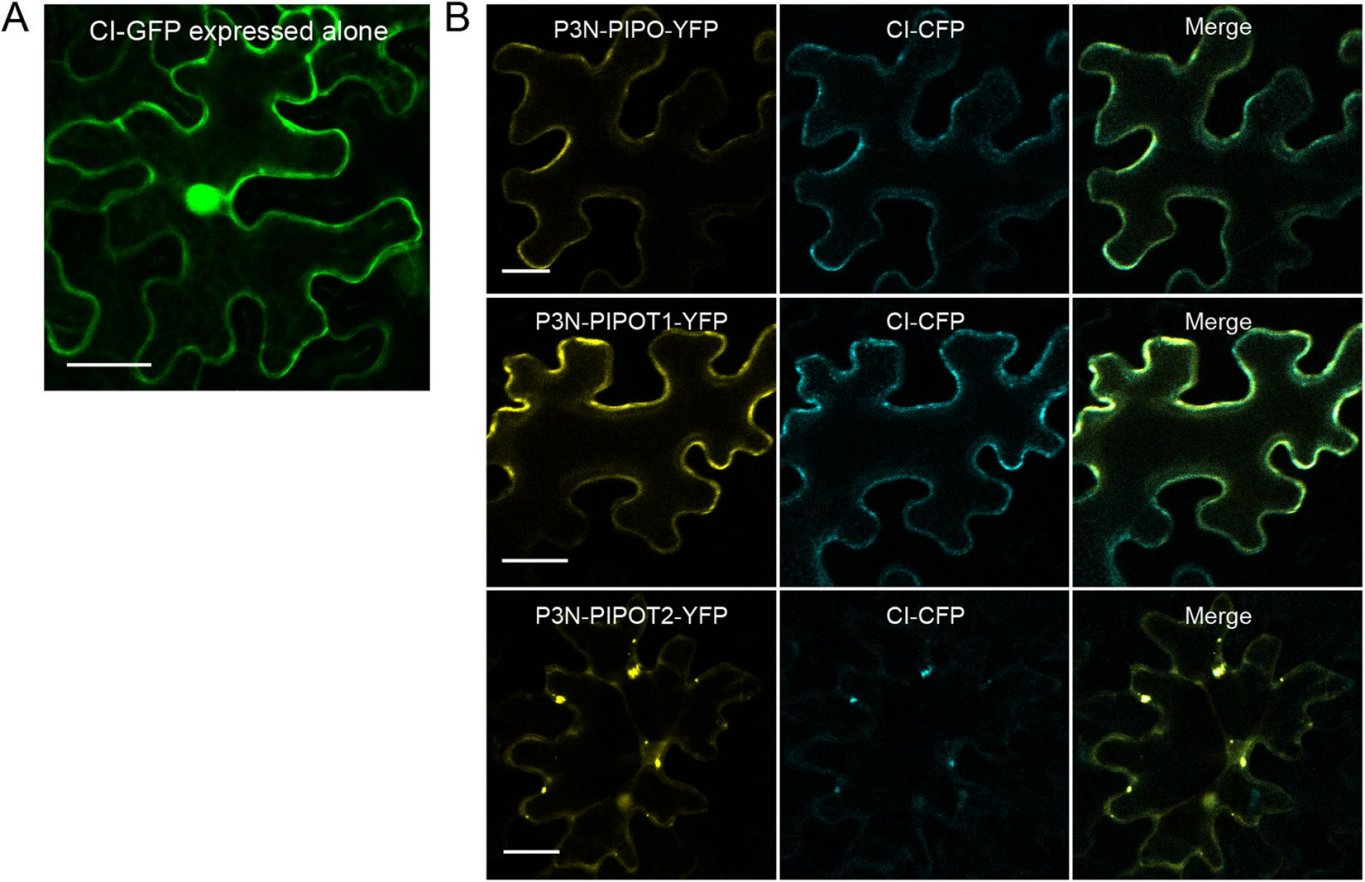
Subcellular localization of SCMV CI in *N*. *benthamiana* leaf cells. (**A**) Localization of CI-GFP expressed alone in leaf cells by 48 hpa. (**B**) Localization of CI-CFP in leaf cells co-expressing P3N-PIPO-YFP, P3N-PIPOT1-YFP or P3N-PIPOT2-YFP. The images were taken by 48 hpa. Scale bars, 25 μm.

Functional P3N-PIPOs could recruit CI to the PD openings, where CIs form conical structures to facilitate the cell-to-cell movement of potyviruses, such as TuMV, TVBMV and TEV^21, 25, 26, 36–38^. The biological functions of the truncated SCMV P3N-PIPOs were indicated by their localization to the PM or PD. Thus, we conducted transient expression assays to investigate if the wild-type P3N-PIPO or truncated P3N-PIPOs could recruit CI to the PM. SCMV CI was fused with GFP or cyan fluorescent protein (CFP), while SCMV P3N-PIPO and the truncated P3N-PIPOs (P3N-PIPOT1 and P3N-PIPOT2) were each fused with YFP. Then, CI-CFP was co-expressed in *N*. *benthamiana* leaves with P3N-PIPO-YFP, P3N-PIPOT1-YFP or P3N-PIPOT2-YFP. The agroinfiltrated leaves were sampled and subjected to confocal observations at 48 hpa. The green fluorescence of the expressed CI-GFP was distributed in the cytoplasm and nucleus when expressed alone (Fig. 4A). The cyan fluorescence of the expressed CI-CFP coincidently overlapped with the yellow fluorescence of the expressed P3N-PIPO-YFP, P3N-PIPOT1-YFP or P3N-PIPOT2-YFP (Fig. 4B). Thus, the wild-type P3N-PIPO and the truncated P3N-PIPOs (P3N-PIPOT1 and P3N-PIPOT2) could recruit CI to the PM.

### SCMV P3N-PIPO facilitates its own cell-to-cell movement

**Figure 5:**
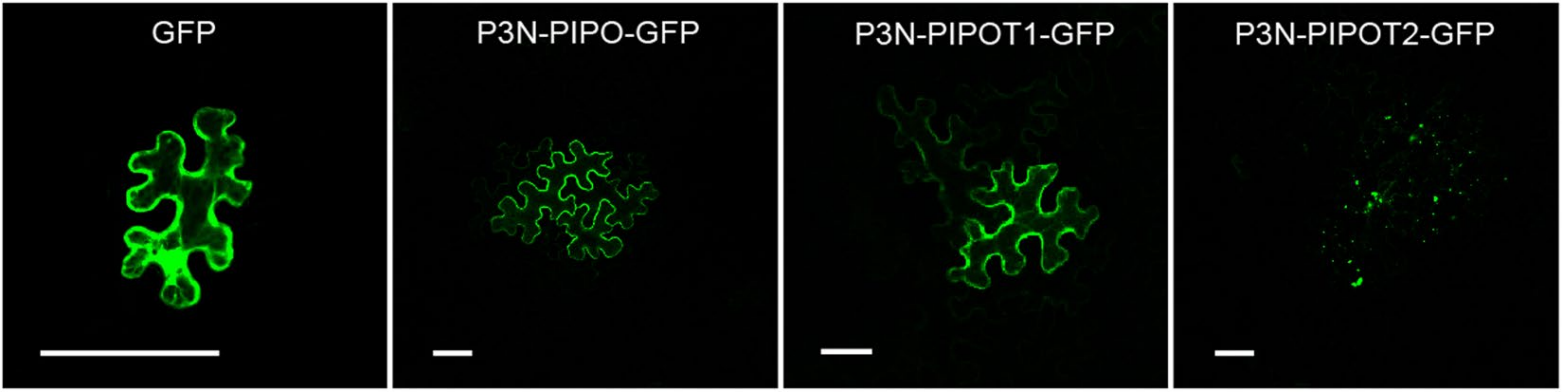
Intercellular movement of wild type P3N-PIPO, P3N-PIPOT1 and P3N-PIPOT2 in *N*. *benthamiana* leaf cells by transient expression. Cell-to-cell movement of GFP, P3N-PIPO-GFP, P3N-PIPOT1-GFP or P3N-PIPOT2-GFP in leaf epidermal cells. Fluorescence of P3N-PIPO-GFP, P3N-PIPOT1-GFP and P3N-PIPOT2-GFP was observed in the initially infected cell and in 2 or more of the surrounding cells compared with GFP expressed alone. Scale bars, 50 μm.

Many viral MPs, such as the putative MP, P3N-PIPO, of TuMV (*Potyvirus*), the MP of *Cauliflower mosaic virus* (*Caulimoviru*s), the 30-kD proteins of *Turnip vein clearing virus* (*Tobamovirus*) and *Tobacco mosaic virus* (*Tobamovirus*), and the MP of *Cabbage leaf curl virus* (*Begomoviruses*) can move to neighbouring cells by themselves^4, 22, 39, 40^. Therefore, we tested the ability of SCMV P3N-PIPO and the truncated P3N-PIPOs to move from cell to cell. *A*. *tumefaciens* cells independently harbouring the GFP, wild-type P3N-PIPO-GFP and the truncated P3N-PIPOs P3N-PIPOT1-GFP and P3N-PIPOT2-GFP were suspended in induction buffer and adjusted to an optical density at 600 nm (OD_600_) = 0.2. Then, the induction buffer was further diluted at a ratio of 1:5,000 to ensure that the initial transfection occurred in isolated foci of a single cell to allow the assessment of cell-to-cell movement. By 48 hpa, the *N*. *benthamiana* leaves agroinfiltrated with GFP showed no cell-to-cell movement (Fig. 5), while those containing P3N-PIPO-GFP, P3N-PIPOT1-GFP and P3N-PIPOT2-GFP showed green fluorescence in clusters of ≥2 epidermal cells (Fig. 5, Table 1). Thus, like the wild-type P3N-PIPO, the truncated P3N-PIPOs could move into neighbouring cells.

**Table 1:** Cell-to-cell trafficking of P3N-PIPO, P3N-PIPOT1 and P3N-PIPOT2 in leaf epidermis of *N*. *benthamiana*.

Injection	Total clusters observed	Clusters with ≥2 cells observed	% of total	*P*-value
GFP	53	6	11.3%	
P3N-PIPO-GFP	60	34	56.7%	<0.001
P3N-PIPOT1-GFP	52	20	38.5%	<0.001
P3N-PIPOT2-GFP	62	28	45.2%	<0.001

*P*-values were calculated using unpaired two-tailed Student t-test.

## Discussion

P3N-PIPOs have been intensively studied in several potyviruses, such as TuMV^21, 22^, TEV^21^, TVBMV^26^ and SMV^41^. The targeting of P3N-PIPOs of TuMV and TEV to the PD was confirmed by plasmolysis^21^. Here, we cloned the coding sequence of P3 from SCMV and obtained SCMV P3N-PIPO by fusion PCR. Transient expression assays showed that SCMV P3N-PIPO localized preferentially to the PM compared with the PD (Fig. 1A,B), which was further confirmed by a plasmolysis assay (Fig. 1C). Thus, the subcellular localization of P3N-PIPO is virus-specific and host-dependent. To identify the domain contributing to PM localization, we generated truncated SCMV P3N-PIPOs. Like the wild-type P3N-PIPO, P3N-PIPOT1 localized to the PM, while P3N-PIPOT2 and PIPOT3 localized to the PD and the PM andalso aggregated in cytoplasm. This suggests that SCMV P3N-PIPO localization to the PD is mediated by the 19-amino acid region at the N-terminus of PIPO. P3N, PIPOT1 and PIPOT2 showed no distinct subcellular localizations. Thus, we speculate that P3N-PIPO localization to the PM is mediated by both P3N and PIPO, indicating that the conformation of P3N-PIPO may play a key role in localization to the PM.

Post-translation modifications, such as glycosylphosphatidylinositol (GPI)-anchoring^42^, palmitoylation^43, 44^ and myristoylation^22, 45^, may also contribute to protein PM localization through membrane rafts. No putative GPI anchor, cleavage sites or myristoylation sites were found using PredGPI^46^ and GPS-Lipid^47^. However, one putative palmitoylation site, Cys180, in SCMV P3N-PIPO was predicted with a score of 4.517. Further investigation of *Potyvirus* members showed that most of their P3N-PIPOs possessed putative palmitoylation sites (Supplementary Table S1). The lipid composition of membrane rafts is very similar to that of PD, and all of the PD localization proteins, display several putative palmitoylation sites^48^. We speculated that the palmitoylation of wild-type P3N-PIPO and truncated P3N-PIPOs contributed to their localizations to membrane rafts. However, further experiments need to confirm this.

Latrunculin B (Lat B) and Brefeldin A (BFA) interfere with cellular processes and have been used to investigate viral intracellular trafficking^21, 26^. Lat B is a chemical inhibitor of actin polymerization^34^. BFA impairs the vesicular transport between the endoplasmic reticulum and Golgi^35^. In this study, leaves of *N*. *benthamiana* were treated with Lat B and BFA independently, and were observed by confocal microscopy. SCMV P3N-PIPO’ localization to the PM or PD was independent of the actin cytoskeleton, while intracellular trafficking required the early secretory pathway (Supplementary Fig. S1). These findings are consistent with previous reports on the P3N-PIPO of TuMV, TEV and TVBMV^21, 26^.

Plant viruses cannot establish infections without interacting with the host factors^49–51^. AtPCaP1, a membrane raft-docking protein^45^, was identified through an *A*. *thaliana* screening to mediate TuMV P3N-PIPO’s localization to the PD by direct interaction, and a mutation of *AtPCaP1* dramatically decreased TuMV cell-to-cell movement^22^. PIPO, but not P3N, determines the interaction between TuMV P3N-PIPO and AtPCaP1^22^. NbDREPP also interacts with TVBMV P3N-PIPO, and the interaction was determined by the 17-amino acid domain on the C-terminus of PIPO^26^. In the present study, we provide evidence that wild-type SCMV P3N-PIPO and P3N-PIPOT1, but not P3N-PIPOT2, interact with ScPCaP1. Thus, the 63-amino acid domain on the C-terminus of PIPO is responsible for SCMV P3N-PIPO’s interaction with ScPCaP1.

BiFC assays showed that SCMV P3N-PIPO and P3N-PIPOT1 interact with ScPCaP1 on the PM in the epidermal cells of *N*. *benthamiana* (Fig. 3B), which is consistent with their PM localization when expressed alone (Fig. 2B). However, P3N-PIPOT2 could localize to the PD without interacting with ScPCaP1. Thus, we hypothesized that interactions with ScPCaP1 contribute to SCMV P3N-PIPO’s localization to the PM, but do not determine SCMV P3N-PIPO’s localization to the PD. This was indirectly confirmed by interfering with the actomyosin system using the Lat B treatment assay, in which the targeting of TVBMV P3N-PIPO to the PD was not impaired, but that of NbDREPP was impaired^26^. Moreover, PCaP1 is not the only protein that interacts with P3N-PIPO. In total, 54 proteins, including glucan endo-1,3-β glucosidase (β-1,3-glucanase), identified from soybean (*Glycine max*) interact with SMV P3N-PIPO^41^. The β-1,3-glucanase localizes to the PD through GPI^52, 53^. Thus, it is possible that P3N-PIPO localizes to the PD by interacting with β-1,3-glucanase or other unknown proteins that are localized to the PD. Additionally, the principle function of PCaP1 may be the regulation of Ca^2+^ concentrations to activate the β-1,3-glucanases’ ability to degrade callose, thus dilating the PD to facilitate viral cell-to-cell movement^3^.

CI recruitment to PD and cell-to-cell movement are the intrinsic capacities of potyviral P3N-PIPO^20–22, 26, 38^. Here, the truncated SCMV P3N-PIPOs, like the wild-type P3N-PIPO, recruited CI to the PM and maybe to the PD (Fig. 4B). Additionally, the truncated SCMV P3N-PIPOs could move through the PD into neighbouring cells (Fig. 5, Table 1). This raises the possibility that the truncated P3N-PIPOs exist in nature. The viral genomes of *Potyvirus* members encode a polyprotein that is then hydrolysed into 10 functional proteins by P1, HC-Pro and NIa-Pro^54, 55^. Is it possible that the P3N-PIPO is subject to hydrolysis? Using an antibody of TuMV PIPO, a 17-kDa unknown protein was identified, which resembles the truncated P3N-PIPO^22^. We believe that SCMV P3N-PIPO can be further hydrolysed. However, further experiments are needed to test this hypothesis.

The P3N-PIPOs of intra- and inter-members of the *Potyviridae* are rich in length polymorphisms^56^. *Potyviridae* members could encode new proteins by polymerase slippage on the GAAAAAA motif^18, 19, 57, 58^. *Clover yellow vein virus* encodes a P3N-ALT at the +1 slippage and the ALT comprising only 7 amino acids. This P3N-ALT mediated the viral cell-to-cell movement without the presence of P3N-PIPOs encoded by −1 slippage^57^. Thus, the selection pressure on P3N-PIPO is not strong. The length polymorphisms of P3N-PIPOs may increase the adaptive abilities of viruses to hosts. Logically, the high levels of P3N-PIPO length polymorphisms indicates the existence of truncated P3N-PIPOs.

In conclusion, we mapped the domain responsible for the localization of SCMV P3N-PIPO to the PM or the PD, which was independent of the actin cytoskeleton, but required the early secretory pathway. We also mapped the domain mediating the interaction with ScPCaP1. The interaction with ScPCaP1 contributes to, but does not determine, SCMV P3N-PIPO localization to the PM or PD.

## Methods

### Plant material and virus

The SCMV strain was provided by the Key Laboratory of Sugarcane Biology and Genetic Breeding, Ministry of Agriculture, Fujian Agriculture and Forestry University, Fuzhou, China and propagated in the sugarcane cultivar *Badila*. Sugarcane plant leaves with typical mosaic symptoms were put into liquid nitrogen immediately after sampling and transferred into a −80 °C freezer for RNA isolation. *N*. *benthamiana* seeds were germinated directly in soil, and plantlets were transferred into culture cups, one plant per cup, at the cotyledon stage. These plants were grown in a climate-controlled chamber with 70% relative humidity at 22 ± 0.5 °C. The photoperiod was 16-h light and 8-h dark under an illumination of 90 μmol/s/m^2^ generated by a fluorescent lamp.

### RNA extraction and RT-PCR

Leaf samples were ground to powder in liquid nitrogen. Then, 1 mL of TriPure Reagent (Roche, Basel, Switzerland) was added to 100 mg of leaf powder. Total RNA was extracted following the manufacturer’s instructions. RNA concentration and quality were determined using the NanoDrop-2000 Spectrophotometer (Thermo Scientific, Wilmington, USA) and electrophoresis. The first strand of cDNA was synthesized from 1 μg of total RNA using a PrimeScript RT-PCR Kit (TaKaRa, Dalian, China) according to the manufacturer’s protocol.

### Gene Cloning

The coding sequence of SCMV CI was cloned by PCR. *ScPCaP1* was cloned by PCR with primers designed based on homologous sequences from sorghum (XP_002453713) and maize (NP_001150000). The SCMV *P3N-PIPO* was cloned by fusion PCR. The P3 cistron was cloned from SCMV using the specific primer pair P3-F/P3-R (Supplementary Table S2). After being confirmed by sequencing, the PCR products were cloned into the vector pMD19-T to generate a template for the cloning of the P3N-PIPO coding sequence. Specific primer pairs were designed based on the P3 cistron to clone the P3N-PIPO coding sequence by fusion PCR (Supplementary Table S2). Primer PIPO-F was designed based on the region containing the motif GA_6_ in which one adenine base was deliberately inserted. Primer P3N-R was reversely complementary to primer PIPO-F. With pMD19-T-P3 as the template, P3N-F/P3N-R was used to amplify *P3N*, and PIPO-F/PIPO-R was used to amplify *PIPO*. *P3N* and *PIPO* PCR products were recovered, purified by electrophoresis and separately concentrated to 400 ng/µL. Then, the two PCR products were mixed at the ratio of 1:1 to form the template for the amplification of *P3N-PIPO* using primer pairs P3N-F/PIPO-R. The *P3N-PIPO* PCR product was purified by agarose gel electrophoresis and recovered. Then, it was cloned into the pMD19-T vector (TaKaRa, Dalian, China) and verified by sequencing.

### Plasmid construction

Gateway technology (Invitrogen, Shanghai, China) was used to generate the constructs for the BiFC and subcellular localization assay in this work. The full-length ORFs or coding sequences of SCMV P3N-PIPO, CI, ScPCaP1 and AtCPK9, as well as those of the truncated P3N-PIPOs, were individually cloned into the pMD19-T vector for preservation and propagation.

Gene sequences were independently recombined into the entry vector pDONR221 (Invitrogen) by the BP reaction, and then the insertions in the resulting pDONR clones were recombined into the destination vectors pEarleygate101, pEarleygate102 and pEarleygate103 and fused with a florescence protein by the LR reaction. *P3N-PIPO* was fused independently with *YFP* and *GFP* to generate P3N-PIPO-YFP and P3N-PIPO-GFP, respectively. *ScPCaP1* was fused independently with *YFP* and *CFP* to generate ScPCaP1-YFP and ScPCaP1-CFP, respectively. *CI* was fused with *CFP* to generate CI-CFP. *AtCPK9* (AT3G20410) was amplified and recombined into pDONR221 and then into the binary destination vector pGWB454, producing CPK9-mRFP.

To dissect the SCMV P3N-PIPO subcellular localization, a series of truncated P3N-PIPO mutations were generated. To identify the SCMV P3N-PIPO domain responsible for PD localization, the truncated *P3N-PIPO* fragments (*P3N*, *PIPO*, *P3N-PIPOT1*, *P3N-PIPOT2*, *PIPOT1*, *PIPOT2* and *PIPOT3*; see Fig. 2) were fused to the *GFP* at the N-terminus of the pEarleygate103 vector. To investigate the co-localization of the truncated P3N-PIPO mutants, P3N-PIPOT1 and P3N-PIPOT2 with CI, *P3N-PIPOT1* and *P3N-PIPOT2* were independently fused to the *YFP* at the N-terminus of the pEarleygate101 vector.

Binary vectors pEarleygate201-YN and pEarleygate202-YC were used to generate constructs for the BiFC assay^31, 50^. *ScPCaP1* was recombined into pEarleygate202-YC to generate ScPCaP1-YC. SCMV *P3N-PIPO* was recombined into pEarleygate202-YN to generate P3N-PIPO-YN. The truncated *P3N-PIPO*s mutants *P3N-PIPOT1* and *P3N-PIPOT2* were individually recombined into pEarleygate202-YN to generate P3N-PIPOT1-YN and P3N-PIPOT2-YN, respectively.

*Sfi*I digestion was used to generate constructs for the Y2H assay. *ScPCaP1* was cloned into pPR3-N to generate construct pPR3-ScPCaP1. *P3N-PIPO*, *P3N-PIPOT1* and *P3N-PIPOT2* were individually cloned into pBT3-STE to generate the constructs pBT3-P3N-PIPO, pBT3-P3N-PIPOT1 and pBT3-P3N-PIPOT2, respectively.

All of the above constructs were verified by sequencing, and all of the primers used in this work are listed in Supplementary Table S2.

### Protein interactions assessed by Y2H and BiFC

For the Y2H assay, the DUALmembrane system (Clontech, Mountain View, CA, USA) was used according to the manufacturer’s protocols. Bait vector pBT3-STE and prey vector pPR3-N harbouring genes to be tested were co-transformed pairwise into the *S*. *cerevisiae NMY51* strain. DDO agar plates and QDO agar plates were used for the yeast cultures. Cells were spread on DDO plates and incubated at 30 °C for 3–5 days after transformation. Colonies grown on DDO plates were suspended in DDO liquid medium to an OD_600_ of 0.6. A 10× dilution series of 5 μL aliquots of co-transformed *NMY51* were spotted onto DDO and QDO agar plates supplemented with X-Gal to test the expression of the LacZ marker. Plates were incubated at 30 °C for 3–5 days. pTSU2-APP and pNubG-Fe65 interact in the Y2H assay and were used as positive controls. pPR3-N and pNubG-Fe65, and pPR3-ScPCaP1 and pBT3-STE, do not form complexes and were used as negative controls.

For the BiFC assays, two fusion constructs were co-agroinfiltrated into *N*. *benthamiana* leaves using needleless syringes. The agroinfiltrated plants were maintained under normal growth conditions for 48 to 72 h.

### Transient expression

The plasmids were transformed into *A*. *tumefaciens* strain *EHA105*. The transformed strain was infiltrated into the leaves of *N*. *benthamiana* using a needleless syringe. Binary vectors were transformed into *A*. *tumefaciens EHA105*. For agroinfiltration, agrobacteria were grown overnight in Luria–Bertani containing the appropriate antibiotics. The agrobacteria were collected by centrifugation and then resuspended in 10 mM MgCl_2_ containing 100 mM acetosyringone. After incubating for a minimum of 2 h at room temperature, the culture was diluted to an OD_600_ of 0.2. *N*. *benthamiana* plants were agroinfiltrated with appropriate agrobacterial cultures, and the agroinfiltrated plants were maintained under normal growth conditions for 12 to 72 h. For plasmolysis, plant tissue was infiltrated with 30% glycerol and viewed immediately. For callose staining, leaves were pressure infiltrated with a 2:3 v/v mixture of 0.1% aniline blue (Fluka) and 1 M glycerol at pH 9.5 for 15 min^32^. All of the experiments were repeated three times, and at least nine plants were used for each treatment.

### Drug treatments

Lat B and BFA treatments were performed to investigate protein intracellular trafficking, as described previously^26, 59^. SCMV P3N-PIPO-GFP was agroinfiltrated in the *N*. *benthamiana* epidermal cells. For Lat B treatment, 5 mM Lat B in 0.1% (v/v) DMSO was infiltrated in the leaves at 40 hpa. Then, the leaves were examined with confocal microscope after 12 h. For BFA treatment, the agroinfiltrated *N*. *benthamiana* leaves were infiltrated with 50 µg/mL BFA in 0.1% (v/v) DMSO at 48 hpa. Then, the leaves were examined with confocal microscope after 3 h. Agroinfiltrated leaves infiltrated with 0.1% (v/v) DMSO were used as controls.

### Confocal microscopy

Agroinfiltrated leaf sections were imaged at room temperature using a Leica SP8 X inverted confocal microscope with an Argon laser (Leica, Wetzlar, Germany). GFP was excited at 488 nm, and the emitted light was captured at 505–555 nm. Chlorophyll autofluorescence was excited at 552 nm, and the emitted light was captured at 650–680 nm. CFP was excited at 442 nm, and the emitted light was captured at 450–500 nm. mCherry and mRFP were excited at 552 nm, and the emitted light was captured at 590–630 nm. YFP was excited at 514 nm, and the emitted light was captured at 530–590 nm. Aniline blue was excited as 405 nm, and the emitted light was captured at 460–500 nm. Images were captured digitally and processed using the Leica Application Suite Advanced Fluorescence Lite (LAS AF version: 2.6.3 build 8173). Generally, 50 to 80 cells were examined for each experiment.

### Bioinformatics analysis

Protein post-translation modifications, such as GPI anchor and cleavage sites, and myristoylation and palmitoylation sites, were predicted using the online software PredGPI (http://gpcr.biocomp.unibo.it/predgpi/pred.htm) and GPS-Lipid (http://lipid.biocuckoo.org/webserver.php), respectively^46, 47^.

## Electronic supplementary material

Supplementary Information

## References

[1] Boevink P, Oparka KJ (2005). Virus-host interactions during movement processes. Plant Physiol.

[2] Verchot-Lubicz J (2010). Varied movement strategies employed by triple gene block-encoding viruses. Mol Plant Microbe Interact.

[3] De Storme N, Geelen D (2014). Callose homeostasis at plasmodesmata: molecular regulators and developmental relevance. Front Plant Sci.

[4] Lucas WJ (2006). Plant viral movement proteins: agents for cell-to-cell trafficking of viral genomes. Virology.

[5] Maule AJ, Havelda Z (2008). *In situ* detection of plant viruses and virus-specific products. Methods Mol Biol.

[6] Ueki S, Citovsky V (2011). To gate, or not to gate: regulatory mechanisms for intercellular protein transport and virus movement in plants. Mol Plant.

[7] Olesinski AA (1996). Tissue-Specific Expression of the Tobacco Mosaic Virus Movement Protein in Transgenic Potato Plants Alters Plasmodesmal Function and Carbohydrate Partitioning. Plant Physiol.

[8] Tzfira T, Rhee Y, Chen MH, Kunik T, Citovsky V (2000). Nucleic acid transport in plant-microbe interactions: the molecules that walk through the walls. Annu Rev Microbiol.

[9] Kawakami S, Watanabe Y, Beachy RN (2004). Tobacco mosaic virus infection spreads cell to cell as intact replication complexes. Proc Natl Acad Sci U S A.

[10] Hofmann C, Sambade A, Heinlein M (2007). Plasmodesmata and intercellular transport of viral RNA. Biochem Soc Trans.

[11] Amari K, Lerich A, Schmitt-Keichinger C, Dolja VV, Ritzenthaler C (2011). Tubule-guided cell-to-cell movement of a plant virus requires class XI myosin motors. PLoS Pathog.

[12] Jackson AO, Lim HS, Bragg J, Ganesan U, Lee MY (2009). Hordeivirus replication, movement, and pathogenesis. Annu Rev Phytopathol.

[13] Lim HS (2009). Subcellular localization of the barley stripe mosaic virus triple gene block proteins. J Virol.

[14] Tilsner J (2013). Replication and trafficking of a plant virus are coupled at the entrances of plasmodesmata. J Cell Biol.

[15] Urcuqui-Inchima S, Haenni AL, Bernardi F (2001). Potyvirus proteins: a wealth of functions. Virus Res.

[16] Riechmann JL, Lain S, Garcia JA (1992). Highlights and prospects of potyvirus molecular biology. J Gen Virol.

[17] Chung BY, Miller WA, Atkins JF, Firth AE (2008). An overlapping essential gene in the Potyviridae. Proc Natl Acad Sci USA.

[18] Olspert A, Carr JP, Firth AE (2016). Mutational analysis of the Potyviridae transcriptional slippage site utilized for expression of the P3N-PIPO and P1N-PISPO proteins. Nucleic Acids Res.

[19] Olspert A, Chung BY, Atkins JF, Carr JP, Firth AE (2015). Transcriptional slippage in the positive-sense RNA virus family Potyviridae. EMBO Rep.

[20] Carrington JC, Jensen PE, Schaad MC (1998). Genetic evidence for an essential role for potyvirus CI protein in cell-to-cell movement. Plant J.

[21] Wei T (2010). Formation of complexes at plasmodesmata for potyvirus intercellular movement is mediated by the viral protein P3N-PIPO. PLoS Pathog.

[22] Vijayapalani P, Maeshima M, Nagasaki-Takekuchi N, Miller WA (2012). Interaction of the trans-frame potyvirus protein P3N-PIPO with host protein PCaP1 facilitates potyvirus movement. PLoS Pathog.

[23] Dolja VV, Haldeman R, Robertson NL, Dougherty WG, Carrington JC (1994). Distinct functions of capsid protein in assembly and movement of tobacco etch potyvirus in plants. EMBO J.

[24] Grangeon R (2013). 6K2-induced vesicles can move cell to cell during *turnip mosaic virus* infection. Front Microbiol.

[25] Roberts IM, Wang D, Findlay K, Maule AJ (1998). Ultrastructural and temporal observations of the potyvirus cylindrical inclusions (Cls) show that the Cl protein acts transiently in aiding virus movement. Virology.

[26] Geng C (2015). Developmentally regulated plasma membrane protein of *Nicotiana benthamiana* contributes to potyvirus movement and transports to plasmodesmata via the early secretory pathway and the actomyosin system. Plant Physiol.

[27] Wen RH, Hajimorad MR (2010). Mutational analysis of the putative pipo of soybean mosaic virus suggests disruption of PIPO protein impedes movement. Virology.

[28] Choi IR, Horken KM, Stenger DC, French R (2005). An internal RNA element in the P3 cistron of *Wheat streak mosaic virus* revealed by synonymous mutations that affect both movement and replication. J Gen Virol.

[29] Wu L, Zu X, Wang S, Chen Y (2013). *Sugarcane mosaic virus* – Long history but still a threat to industry. Crop Protection.

[30] Xu DL, Park JW, Mirkov TE, Zhou GH (2008). Viruses causing mosaic disease in *sugarcane* and their genetic diversity in southern China. Arch Virol.

[31] Zhai Y (2015). *Sugarcane* Elongin C is involved in infection by *sugarcane mosaic disease* pathogens. Biochem Biophys Res Commun.

[32] Simpson C, Thomas C, Findlay K, Bayer E, Maule AJ (2009). An Arabidopsis GPI-anchor plasmodesmal neck protein with callose binding activity and potential to regulate cell-to-cell trafficking. Plant Cell.

[33] Benetka W (2008). Experimental testing of predicted myristoylation targets involved in asymmetric cell division and calcium-dependent signalling. Cell Cycle.

[34] Morton WM, Ayscough KR, McLaughlin PJ (2000). Latrunculin alters the actin-monomer subunit interface to prevent polymerization. Nat Cell Biol.

[35] Nebenfuhr A, Ritzenthaler C, Robinson DG (2002). Brefeldin A: deciphering an enigmatic inhibitor of secretion. Plant Physiol.

[36] Rodríguez-Cerezo E (1997). The Coat and Cylindrical Inclusion Proteins of a Potyvirus Are Associated with Connections between Plant Cells. Virology.

[37] Roberts IM, Wang D, Thomas CL, Maule AJ (2003). *Pea seed-borne mosaic virus* seed transmission exploits novel symplastic pathways to infect the pea embryo and is, in part, dependent upon chance. Protoplasma.

[38] Deng P, Wu Z, Wang A (2015). The multifunctional protein CI of potyviruses plays interlinked and distinct roles in viral genome replication and intercellular movement. Virol J.

[39] Lewis JD, Lazarowitz SG (2010). *Arabidopsis* synaptotagmin SYTA regulates endocytosis and virus movement protein cell-to-cell transport. Proc Natl Acad Sci USA.

[40] Uchiyama A (2014). The *Arabidopsis* synaptotagmin SYTA regulates the cell-to-cell movement of diverse plant viruses. Front Plant Sci.

[41] Song P (2016). Identification for soybean host factors interacting with P3N-PIPO protein of *Soybean mosaic virus*. Acta Physiologiae Plantarum.

[42] Galian C, Bjorkholm P, Bulleid N, von Heijne G (2012). Efficient glycosylphosphatidylinositol (GPI) modification of membrane proteins requires a C-terminal anchoring signal of marginal hydrophobicity. J Biol Chem.

[43] Gui J, Zheng S, Shen J, Li L (2015). Grain setting defect1 (GSD1) function in rice depends on S-acylation and interacts with actin 1 (OsACT1) at its C-terminal. Front Plant Sci.

[44] Kumar M (2016). S-Acylation of the cellulose synthase complex is essential for its plasma membrane localization. Science.

[45] Nagasaki N, Tomioka R, Maeshima M (2008). A hydrophilic cation-binding protein of *Arabidopsis thaliana*, AtPCaP1, is localized to plasma membrane via *N*-myristoylation and interacts with calmodulin and the phosphatidylinositol phosphates PtdIns(3,4,5)*P*3 and PtdIns(3,5)*P*2. FEBS J.

[46] Pierleoni A, Martelli PL, Casadio R (2008). PredGPI: a GPI-anchor predictor. BMC Bioinformatics.

[47] Xie Y (2016). GPS-Lipid: a robust tool for the prediction of multiple lipid modification sites. Sci Rep.

[48] Grison MS (2015). Specific membrane lipid composition is important for plasmodesmata function in *Arabidopsis*. Plant Cell.

[49] Wittmann S, Chatel H, Fortin MG, Laliberte JF (1997). Interaction of the viral protein genome linked of *turnip mosaic potyvirus* with the translational eukaryotic initiation factor (iso) 4E of Arabidopsis thaliana using the yeast two-hybrid system. Virology.

[50] Leonard S (2000). Complex formation between potyvirus VPg and translation eukaryotic initiation factor 4E correlates with virus infectivity. J Virol.

[51] Wang A (2015). Dissecting the molecular network of virus-plant interactions: the complex roles of host factors. Annu Rev Phytopathol.

[52] Levy A, Erlanger M, Rosenthal M, Epel BL (2007). A plasmodesmata-associated beta-1,3-glucanase in *Arabidopsis*. Plant J.

[53] Benitez-Alfonso Y (2013). Symplastic intercellular connectivity regulates lateral root patterning. Dev Cell.

[54] Kim DH (1995). Expression, purification, and identification of a novel self-cleavage site of the Nla C-terminal 27-kDa protease of *turnip mosaic potyvirus* C5. Virology.

[55] Adams MJ, Antoniw JF, Fauquet CM (2005). Molecular criteria for genus and species discrimination within the family Potyviridae. Arch Virol.

[56] Hillung J, Elena SF, Cuevas JM (2013). Intra-specific variability and biological relevance of P3N-PIPO protein length in potyviruses. BMC Evol Biol.

[57] Hagiwara-Komoda Y (2016). Truncated yet functional viral protein produced via RNA polymerase slippage implies underestimated coding capacity of RNA viruses. Sci Rep.

[58] Mingot A (2016). The P1N-PISPO trans-Frame Gene of Sweet Potato Feathery Mottle Potyvirus Is Produced during Virus Infection and Functions as an RNA Silencing Suppressor. J Virol.

[59] Brandizzi F, Snapp EL, Roberts AG, Lippincott-Schwartz J, Hawes C (2002). Membrane protein transport between the endoplasmic reticulum and the Golgi in tobacco leaves is energy dependent but cytoskeleton independent: evidence from selective photobleaching. Plant Cell.

