# Supplementary material for: Dissecting the Molecular Mechanism of the Subcellular Localization and Cell-to-cell Movement of the Sugarcane mosaic virus P3N-PIPO
Source: Sci Rep. 2017 Aug 29;7:9868. doi: 10.1038/s41598-017-10497-6 (PMC5575073; doi:10.1038/s41598-017-10497-6)
Supplement: Supplementary file 1 — Supplementary Information [file 41598_2017_10497_MOESM1_ESM.pdf]

Dissecting the molecular mechanism of the subcellular localization and cell-to-cell movement of the *Sugarcane mosaic virus* P3N-PIPO

Guangyuan Cheng[1], Meng Dong[1], Qian Xu[1], Lei Peng[1], Zongtao Yang[1], Taiyun Wei[2]*, Jingsheng Xu[1]*

[1]Key Laboratory of Sugarcane Biology and Genetic Breeding, Ministry of Agriculture, Fujian Agriculture and Forestry University, Fuzhou 350002, Fujian, China.

[2]State Key Laboratory of Ecological Pest Control for Fujian and Taiwan Crops, Institute of Plant Virology, Fujian Agriculture and Forestry University, Fuzhou 350002, Fujian, China.

*Corresponding author: Jingsheng Xu, e-mail: xujingsheng@126.com; Taiyun Wei, email: weitaiyun@fafu.edu.cn.

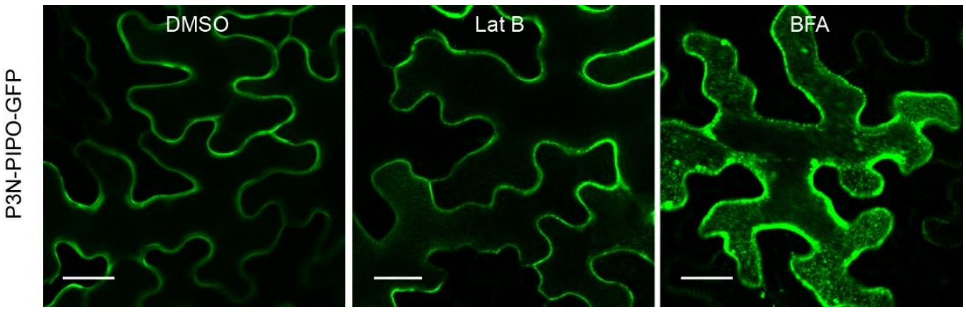

Figure S1. Lat B and BFA treatments interfering the intracellular trafficking of SCMV P3N-PIPO. SCMV P3N-PIPO was expressed in the *N. benthamiana* epidermal cells treated with 0.1% (v/v) DMSO or 50 μg/mL BFA or 5 μM Lat B. Images were captured at 48 hpa. Scale bars, 25 μm.

Supplementary Table S1. Primers used in this study

Primer name	Primer sequence (5'→3')	Strategy
P3-F	GTTGATCATGAGAGCAAGTCAATGC	For gene clone
P3-R	AAATCTTGATTTGTATCAAAATC	
CI-F	GATTAGCACAATTGAGCAGAATG	
CI-R	CCATTCCTTGGTGAATAACTGTG	
ScPCaP1-F	ATAAACATCAGGGGCGGCGT	
ScPCaP1-R	GAGCAACAAAAACACATCGT	
P3N-F	GGCACCCTGACACAGCAAACG	
P3N-R	TAATTGATCTACATAACTTTTTTTCCGTTAAAGTGTATAAGCGTTCGTC	
PIPO-F	GACGAACGCTTATACACTTTAACGGAAAAAAAGTTATGTAGATCAATTA	
PIPO-R	TTATCGCAAAAACTGTGGCGG	
AttB-PCaP1-F	GGGGACAAGTTTGTACAAAAAAGCAGGCTTCATGGACATCTGGAAGTCCAAGG	Construction of vectors for BiFC and subcellular localization
AttB-PCaP1-R	GGGGACCACTTTGTACAAGAAAGCTGGGTCGGCCTTGGGTGTCTCCTCC	
AttB-CI-F	GGGGACAAGTTTGTACAAAAAAGCAGGCTTCAGTGTAGATGTTGATGA	
AttB-CI-R	GGGGACCACTTTGTACAAGAAAGCTGGGTCTTGGTGAATGACTGTGTTTA	
AttB-P3N-PIPO-F	GGGGACAAGTTTGTACAAAAAAGCAGGCTTCGGCACCCTGACACAGCAA	
AttB-P3N-PIPO-R	GGGGACCACTTTGTACAAGAAAGCTGGGTCTCGCAAAAACTGTGGCGG	
AttB-P3N-PIPOT1-R	GGGGACCACTTTGTACAAGAAAGCTGGGTCGATATATTGTAAACAGCGCC	
AttB-P3N-PIPOT2-R	GGGGACCACTTTGTACAAGAAAGCTGGGTCCCATAATATGACAACTCTGCC	
AttB-P3N -R	GGGGACCACTTTGTACAAGAAAGCTGGGTCCGTTAAAGTGTATAAG	
AttB-PIPO-F	GGGGACAAGTTTGTACAAAAAAGCAGGCTTCATGGAAAAAAAGTTATGTAGATC	
AttB-PIPOT1-F	GGGGACAAGTTTGTACAAAAAAGCAGGCTTCATGAAAATTTTCAGCAATATGG	
AttB-PIPOT2-F	GGGGACAAGTTTGTACAAAAAAGCAGGCTTCATGAGCTACGCATCTAATATC	
AD-PCaP1-F	ATTAACAAGGCCATTACGGCCATGGACATCTGGAAGTCCAAGG	Construction of vectors for Y2H
AD-PCaP1-R	TTGACTAAGGCCGAGGCGGCCGCTAGGCCTTGGGTGTCTCCTCC	
BD-P3N-PIPO-F	ATTAACAAGGCCATTACGGCCGGCACCCTGACACAGCAA	
BD-P3N-PIPO-R	TTGACTAAGGCCGAGGCGGCCCCTCGCAAAAACTGTGGCGG	
BD-P3N-PIPOT1-R	TTGACTAAGGCCGAGGCGGCCCCGATATATTGTAAACAGCGCC	
BD-P3N-PIPOT2-R	TTGACTAAGGCCGAGGCGGCCCCCCATAATATGACAACTCTGCC	

Supplementary Table S2. Prediction of S-acylation sites of PIPO from members of *Potyvirus*.

Accession number	Virus	Length (aa)	Predicted S-acylation sites	Score	Cutoff
	Sugarcane mosaic virus	82	Cys5	5.071	3.076
			Cys27	4.517	3.076
ANU06082	*Plum pox virus*	102	Cys22	0.951	0.893
YP_006405426	*Pepper veinal mottle virus*	72	Cys9	0.863	0
			Cys18	0.894	0
			Cys56	1.361	0
YP_006393459	*Tobacco etch virus*	99	Cys4	4.335	3.076
			Cys10	16.656	1.983
			Cys53	1.835	0
			Cys85	0.330	0
AGL43557	*Potato virus Y*	76	Cys11	1.578	1.396
			Cys69	1.792	0
YP_006393461	*Pea seed-born mosaic virus*	79	Cys17	0.483	0
YP_006405418	*Tobacco vein banding mosaic virus*	60	Cys56	1.410	0
AMW92445	*Papaya ringspot virus*	73	Cys14	11.809	1.983
			Cys27	1.922	0
			Cys49	1.035	0.893
			Cys58	0.140	0
YP_003587806	*Turnip mosaic virus*	61	Cys22	3.217	1.983
AIZ09254	*Soybean mosaic virus*	76	Cys31	1.887	0
			Cys43	0.263	0
			Cys56	0.593	0
			Cys61	0.497	0
			Cys68	0.556	0
ALS88435	*Johnsongrass mosaic virus*	90	n.d.	n.d.	n.d.
YP_006393471	*Maize dwarf mosaic virus*	80	Cys4	4.093	3.076
			Cys26	0.342	0
			Cys48	0.909	0.893
YP_006395325	*Bean commen mosaic virus*	73	Cys4	4.258	3.076
			Cys22	0.447	0
			Cys42	1.026	0
			Cys53	0.271	0
			Cys55	0.327	0
			Cys60	0.256	0
			Cys67	0.198	0
YP_006423944	*Freesia mosaic virus*	84	Cys4	6.222	3.076
			Cys71	0.200	0
YP_006393476	*Bean yellow vein virus*	79	Cys37	0.840	0
			Cys66	2.787	2.293
			Cys72	0.564	0
			Cys75	1.302	1.072

YP_006395313	*Clover yellow vein virus*	60	Cys45	0.717	0
AKI32474	*Potato virus A*	94	Cys36	1.458	1.396
			Cys37	2.372	1.396
			Cys56	2.018	1.991
			Cys72	2.462	2.293
YP_006395319	*Potato virus V*	69	n.d.	n.d.	n.d.
KM025043	*Sorghum mosaic virus*	81	Cys27	0.543	0
YP_006424010	*Wild potato mosaic virus*	69	n.d.	n.d.	n.d.
ANK58177	*Yam mild mosaic virus*	68	Cys5	3.826	3.076
			Cys49	3.767	3.076
			Cys53	0.693	0
			Cys68	0.337	0
YP_006395329	*Onion yellow dwarf virus*	75	Cys21	2.479	2.293
			Cys44	0.406	0
			Cys49	0.843	0
			Cys61	0.441	0
YP_006395331	*Lily mottle virus*	62	Cys9	2.077	1.396
			Cys53	1.124	0
			Cys55	0.398	0
YP_006395332	*Beet mosaic virus*	115	Cys4	4.599	3.076
			Cys23	0.946	0.893
			Cys47	0.334	0
			Cys71	0.714	0
			Cys26	1.501	1.396
			Cys107	1.294	1.072
			Cys108	3.865	3.076
ANF99507	*Moroccan watermelon mosaic virus*	60	Cys22	1.115	1.079
			Cys60	1.605	0
YP_006395338	*Pennisetum mosaic virus*	81	Cys26	5.310	3.076
			Cys48	0.609	0
			Cys79	3.518	1.983
YP_006395351	*East Asian Passiflora virus*	75	Cys4	5.080	3.076
			Cys22	1.795	1.079
			Cys33	0.672	0
			Cys42	2.092	1.991
			Cys53	0.691	0
			Cys60	0.289	0
YP_006401479	*Pepper severe mosaic virus*	74	Cys4	5.820	3.076
			Cys34	0.854	0
			Cys55	0.156	0
			Cys62	5.003	3.076
			Cys63	3.668	1.396
			Cys64	2.508	1.396
YP_006401477	*Konjac mosaic virus*	81	Cys23	2.959	2.293

			Cys51	0.159	0
YP_006401478	*Dnphne mosaic virus*	72	Cys27	1.398	1.396
			Cys42	0.296	0

Note: n.d. stands for not discovered.
